# “BUILDING A PLANE WHILE IT’S TAKING OFF”: PERSPECTIVES OF HOSPICE PROVIDERS FROM GROUND ZERO OF THE COVID-19 CRISIS IN NYC

**DOI:** 10.1093/geroni/igad104.0624

**Published:** 2023-12-21

**Authors:** Daniel David, Jonelle Boafo, Patricia Kim, Laura Moreines, Emily Franzosa, Dena Schulman-Green, Abraham Brody, Melissa Aldridge

**Affiliations:** NYU Rory Meyers College of Nursing, New York City, New York, United States; Rory Meyers College of Nursing, New York City, New York, United States; Icahn School of Medicine at Mount Sinai, New York City, New York, United States; New York University, New York City, New York, United States; Icahn School of Medicine at Mount Sinai, New York City, New York, United States; NYU, College of Nursing, New York City, New York, United States; NYU Rory Meyers College of Nursing, New York City, New York, United States; Icahn School of Medicine at Mount Sinai, New York City, New York, United States

## Abstract

The provision of care to older adults with serious illness through home hospice visits was extremely challenging for interdisciplinary teams during the COVID-19 pandemic in NYC. We interviewed 20 interdisciplinary providers (nurses, managers, administrators, physicians, medical directors, social workers, and clergy) from three organizations that provided end-of-life care in-person and through telehealth to older adults and their caregivers in NYC. Qualitative content analysis was used to identify themes, categorize findings, and identify important adaptations that were required to provide hospice care during the pandemic. Findings reveal how organizations, providers, patients, and families contended with fear, communicated challenges, and adapted to an ever-changing regulatory landscape. The experience of providing care was markedly different for those who were required to deliver care in person vs. by telehealth. Difficult care choices were made impacting access and the services offered by hospice organizations. Salient themes included: organizational challenges – “building a plane while it’s taking off” (e.g., implementing institutional safety protocols with imperfect COVID-19 information to a wary staff), patient/family challenges – “you aren’t coming in my home” (e.g., inability to enroll patients into hospice without a face-to-face visit), and provider challenges – “some things you can’t do through telehealth.” (e.g., telehealth is a vastly inferior medium to teach isolated family caregivers how to provide end-of-life support to dying family members). These findings provide an opportunity to consider the unique qualities of hospice care, what care can and cannot be effectively delivered through virtual modalities, and what effective adaptations may be sustained long-term.

